# TGF-β secreted by cancer cells-platelets interaction activates cancer metastasis potential by inducing metabolic reprogramming and bioenergetic adaptation

**DOI:** 10.7150/jca.103757

**Published:** 2025-01-13

**Authors:** Chunlian Zhong, Weiyu Wang, Yinyin Yao, Shu Lian, Xiaodong Xie, Judan Xu, Shanshan He, Lin Luo, ZhouZhou Ye, Jiajie Zhang, Mingqing Huang, Guihua Wang, Yanhong Wang, Yusheng Lu, Chengbin Fu

**Affiliations:** 1 Fuzhou Institute of Oceanography, Fujian-Taiwan-Hongkong-Macao Science and Technology Cooperation Base of Intelligent Pharmaceutics, College of Material and Chemical Engineering, Minjiang University, Fuzhou, Fujian 350108, China.; 2 College of Pharmacy, Fujian Key laboratory of Chinese Materia Medical, Fujian University of Traditional Chinese Medicine, Fuzhou, Fujian 350122, China.; 3 College of Chemistry and Chemical Engineering, Fuzhou University, Fuzhou 350116, China.; 4 Heilongjiang Provincial Key Laboratory of Environmental Microbiology and Recycling of Argo-Waste in Cold Region, College of Life Science and Biotechnology, Heilongjiang Bayi Agricultural University, Daqing 163319, China.; 5 Key Laboratory of Low-carbon Green Agriculture in Northeastern China, Ministry of Agriculture and Rural Affairs, China.; 6 Department of Breast Surgery, Fujian Medical University Union Hospital, Fujian Medical University, Fuzhou 350001, China.

**Keywords:** Cancer metastasis, platelet-cancer cells interaction, transforming growth factor-β (TGF-β), metabolic reprogramming, bioenergetic adaptation, epithelial-to-mesenchymal transition (EMT)

## Abstract

Metastasis is the leading cause of cancer-related deaths and poses a treatment challenge. Although studies have shown the importance of epithelial-mesenchymal transition (EMT) and metabolic reprogramming during cancer metastasis, the link between EMT and metabolic reprogramming, as well as the underlying molecular mechanisms by which both mediate cancer cell invasion and metastasis have not been elucidated. Here, we observed that interactions between platelets and cancer cells promote the secretion of TGF-β, thereby initiating EMT, promoting the invasion, and altering the metastatic and metabolic potential of colon cancer cells. TGF-β activates the AKT signaling pathway to enhance HK1 and HK2 expression in cancer cells, leading to increased glucose consumption, ATP production, and precise modulation of cell cycle distribution. In an energy-deficient model induced by oxidative phosphorylation (OXPHOS) inhibition with oligomycin A, TGF-β-induced highly metastatic HCT116 (H-HCT116) cells adapt by upregulating HK expression and glycolytic metabolism, while concurrently decreasing cell proliferation to conserve energy for survival. Mechanistically, H-HCT116 cells regulate cell division rates by downregulating CDK2, CDK4, and Cyclin D1 protein expression and upregulating p21 expression. Furthermore, H-HCT116 cells display enhanced motility, which is linked to increased mitochondrial metabolic activity. These findings indicated that cancer cells-platelets interaction secreted TGF-β activates cancer metastasis potential by inducing metabolic reprogramming and bioenergetic adaptation. The present study provides new insights into the adaptive strategies of highly metastatic cancer cells under adverse conditions and indicates that targeting glycolysis and metabolic reprogramming could serve as a viable approach to prevent cancer metastasis.

## 1. Introduction

Cancer is a significant challenge to the world's public health, with about 20 million new cases to be diagnosed and 9.7 million deaths attributable to the disease in 2022 1. The metastatic spread of cancer remains the primary cause of mortality among patients and poses a significant obstacle in advancing cancer management 2. Colorectal cancer (CRC) is one of the most frequently diagnosed malignancies and the main cause of cancer-related deaths worldwide 3. At the time of diagnosis, a significant number of patients have already progressed to the stage of tumor cell metastasis, and approximately 50% of CRC patients experiencing distant metastases 4. Despite the advancements in therapeutic options that have enhanced the prognosis of patients with CRC, a majority of cancer survivors still face an unfavorable outcome primarily due to metastasis. Hence, it is imperative to investigate the molecular mechanisms underlying colon cancer metastasis and devise preventive strategies.

Extensive evidence indicated that platelet plays an important role in the process of metastasis, as circulating tumor cells (CTCs) evade immune surveillance through interactions with platelets, thereby facilitating their survival and subsequent metastatic dissemination 5, 6. Furthermore, activated platelet release of transforming growth factor β (TGF-β) enhance cancer metastasis by inducing epithelial-mesenchymal transition (EMT) that facilitates cancer cell extravasation and tissue invasion 7-9. EMT represents a key transformation where cells lose epithelial features and gain mesenchymal and stem cell-like characteristics, which enhance invasiveness 10, 11. TGF-β signaling can induce EMT through Smads and PI3K/AKT signaling pathways, thereby activating EMT transcription factors 12-14. The active involvement of PI3K/AKT signaling pathways in promoting invasion by metastatic cancer cells has been extensively researched, including the regulation of EMT-related proteins (such as E-cadherin) and transcription factors 15, 16.

Metabolic reprogramming is one of the hallmarks of cancer, and plays a crucial role in regulating the survival and metastasis of cancer cells 17, 18. Cancer cells usually require much more energy than normal cells to sustain their rapid proliferation. The cell primarily relies on mitochondrial oxidative phosphorylation (OXPHOS) and glycolysis as its main energy metabolism pathways 19, 20. The majority of cancer cells exhibit a high rate of glycolysis, which not only compensates for the increased demand for adenosine 5'-triphosphate (ATP), but also contributes to cell proliferation and survival by modulating signaling pathways and enhancing the biosynthesis of macromolecules, including proteins, nucleic acids and lipids 21-23. The first key enzymes in glycolysis, known as hexokinases (HK), are essential kinases that converts glucose into glucose 6-phosphate 24. Under the influence of an altered cancer microenvironment, metabolic reprogramming facilitates the adaptation of cancer cells to sustain aggressive growth and metastasis through efficient energy acquisition 25. Although studies have shown the importance of EMT and metabolic reprogramming during cancer metastasis, the link between EMT and metabolic reprogramming, as well as the underlying molecular mechanisms by which both mediate cancer cell invasion and metastasis have not been elucidated.

In the present study, we investigated the effects of TGF-β on the cancer cell migration activity, metabolic potential, and proliferation rate. Additionally, we examined the ability of TGF-β-induced high-metastatic cancer cells to adapt to extreme environments (such as inhibition of OXPHOS) and their stress response ability. By exploring the molecular mechanisms of TGF-β-induced metabolic reprogramming and bioenergetic adaptation, this study aims to provide new insights into the adaptive strategies of highly metastatic cancer cells in challenging microenvironments.

## Materials and methods

### Cell culture

The human colorectal cancer cell lines HT29, HCT116, and LoVo were obtained from the Shanghai Cell Bank, Chinese Academy of Sciences. HT29 and HCT116 cells were cultured in McCoy's 5A medium (Sigma-Aldrich, USA) supplemented with 1% fetal bovine serum (FBS, Life Technology, USA), 100 units/mL penicillin (GenView, USA), and 100 μg/mL streptomycin (GenView, USA). LoVo cells were cultured in RPIM1640 medium (Hyclone, USA) supplemented with 1% FBS, 100 units/mL penicillin and 100 μg/mL streptomycin. All cells were cultured at 37°C in a humidified atmosphere containing 5% CO_2_ and harvested when they reached 85% confluence using a solution of 0.25% trypsin (GenView, USA).

### Platelets/cancer cells interplay assay

Blood was collected from healthy volunteers who had not taken any drugs known to affect platelet function for at least 14 days prior to the study. The study was approved by the local ethics committee and conducted in compliance with the Declaration of Helsinki regarding ethical conduct of research involving human subjects. Written informed consent was obtained from each healthy volunteer. Platelets were added to 24-well plates inoculated with HT29 cells, and the positive controls group was treated with ADP-activated platelets. After incubation for 4 hours, the culture medium was collected by centrifuging at 1500×g for 15 min to remove platelets. The TGF-β levels in culture medium were detected using the Human TGF-β Sunny ELISA Kit (Lianke Biotech, Co., Ltd, China) according to the manufacturer's protocol. The protein expression levels of p-Smad in HT29 cells were analyzed by western blot.

### Observation of cell morphology

HT29, HCT116 and LoVo cells were seeded at a density of 1×10^5^ cells per well in a 6-well plate for 24 h. Then the cells were treated with or without 10 ng/mL TGF-β (Peprotech, USA) for various periods (0, 1, 3, 5, 7, and 14 days). Cell morphological changes were observed by microscope (Leica, Germany) and photographed in 10 randomly selected microscopic fields.

### Invasion assay

The effect of TGF-β on the invasive ability of HT29 and HCT116 for various periods (0, 1, 3, 5, and 7 days) was analyzed by the transwell assay 26, 27. Briefly, the transwell culture chambers (24-well, 8 μm pore size, Costar, Corning Incorporated, USA) were placed in a 24-well plate and the upper chambers were coated with Matrigel (BD Biocoat ^TM^, USA) and air-dried. After 1 h incubation at room temperature, chambers were gently washed three times with 1 mL PBS. All cells were treated with 10 ng/mL TGF-β for various periods (0, 1, 3, 5, and 7 days). The day before the transwell assay, these cells were starved overnight with FBS-free but containing 10 ng/mL TGF-β medium t. 25×10^4^ cells were suspended in 250 μL of serum-free medium containing 0.1% BSA (GenView, USA) and placed into the upper chamber of the transwell unit. The lower chambers were added with 750μL of medium containing 20% FBS as a chemoattractant. After 24 h incubation at 37°C in 5% CO_2_, the non-invading cells on the upper surface of the chambers were slowly wiped off using a cotton swab. Cells invaded through the matrix membrane were fixed with 4% (w/v) paraformaldehyde for 30 minutes and stained with 0.1% crystal violet for 30 minutes. A random selection of 10 fields was photographed and counted under the microscope.

### Cell cycle assay

TGF-β (10 ng/mL) was treated on HCT116 cells for different times (0, 1, 3, 5, 7, 14 days) to evaluate the effect on the cell cycle. Oligomycin A (1 μM, Selleck Chemicals, USA) was used to assess the effect of oxidative phosphorylation (OXPHOS) on the cell cycle of HCT116 cells after 7 days of TGF-β treatment. Cells were collected and analyzed for cell cycle distribution by flow cytometry as described 26, 28.

### Glucose metabolism assay

HT29 and HCT116 cells were treated with 10 ng/mL TGF-β for various periods (0, 1, 7 days). These cells sorted by flow cytometry were seeded into 6-well plates (5×10^5^/well) and cultured overnight. The cells were cultured for 48h after replacing the medium with fresh medium. After then, the supernatant was collected and the remaining glucose was determined using the Glucose Assay Kit (Sigma-Aldrich, USA). The remaining glucose was calculated after the sample readings were applied to the glucose standard curve according to the manufacturer's protocol.

### Intracellular ATP level assay

HCT116 and HT29 cells were induced with TGF-β for different times (0, 0.5, 1, 3, 5, 7 14, 21 days) or different concentrations (0, 1, 5, 10, 20, 40 ng/mL). These cells sorted by flow cytometry were seeded into 96-well plates (1×10^4^/well) and cultured overnight. The intracellular ATP level of these cells was determined by the Luminescence method using an ATP Assay Kit (Perkin-Elmer, USA) according to the manufacturer's instructions 29.

### Effects of OXPHOS and oxygen content on intracellular ATP level of H-HCT116 cells

HCT116 cells were induced with or without 10 ng/mL TGF-β for 7 days. These cells sorted by flow cytometry were seeded into 96-well plates (1×10^4^/well) and cultured overnight. Then the medium was replaced with a medium containing 1 μM oligomycin A and 1% FBS. For ATP detection, cells induced with oligomycin A were cultured under different conditions (atmosphere of 5% or 20% O_2_) for various periods (0, 1, 4, 8, 12, 24, 48 h). The intracellular ATP level of these cells was determined by the Luminescence method using an ATP Assay Kit (Perkin-Elmer, USA).

### Effect of TGF-β on Epithelial-to-mesenchymal-transition (EMT) on cancer cells

To examine the effect of TGF-β on EMT, HT29, HCT116, and LoVo cells were seeded in 6-well plates. The cells were treated with a fresh medium containing 10 ng/mL TGF-β for various periods (0, 1, 3, and 7 days). After incubation, the mRNA expression levels of HK1, HK2, ZEB1, ZEB2, SNAIL1, SNAIL2, FN1, VIM, CDH1, HIF1, CD133, TWIST, MMP2, TIMP, and β-actin mRNA were analyzed by quantitative reverse transcription-quantitative polymerase chain reaction (qRT-PCR), all primer sequences for PCR analysis are presented in **Supplementary Table 1**. The protein expression levels of AKT, p-AKT, E-cadherin, and β-actin were analyzed by western blot.

### Effects of OXPHOS on Cyclin of H-HCT116 cells

To examine the effect of TGF-β on OXPHOS, HCT116 cells were induced with or without 10 ng/mL TGF-β for 7 days. These cells were seeded at a density of 1×10^5^ cells per well in a 6-well plate overnight. Then the cells were treated with 1 μM oligomycin A for various periods (0, 1, 4, 8, 24,48 h). After incubation, cells were lysed by RIPA, and the protein expression levels of HK1, HK2, pRB (S807/811), pRB (S795), pChk1, pChk2, p18, p21, p27, CDK2, CDK4, CDK6, Cyclin D1, Cyclin D3, β-actin were analyzed by western blot.

### Effects of TGF-β on cytoskeleton and mitochondrial distribution

HCT116 cells were cultured on a confocal dish with or without 10 ng/mL TGF-β for 7 days. Subsequently, the medium was replaced with a medium containing 1 μM oligomycin A and 1% FBS for 24 h. Following incubation, the medium was removed from the dish and replaced with 250 nM Mito-Tracker Red CMXRos (Life Technology, USA). After incubation for 30 min, these cells were washed with PBS and fixed with 4% paraformaldehyde at room temperature for 10 min. After fixation, cells were washed with PBS and then permeabilized with 0.1% Triton X-100 in PBS at room temperature for 5 minutes. This was followed by blocking with 5% bovine serum albumin (BSA, GenView, USA) in PBS for a duration of 30 minutes. Subsequently, the cells were incubated with Phallotoxins-Alexa Fluor 488 antibodies (Life Technology, USA) at room temperature for a period of 20 minutes. Confocal dishes were then mounted using fluorescent mounting media containing DAPI (Beyotime, China). Images were immediately acquired using a TCS SP8 confocal microscope (Leica, Germany), employing consistent acquisition parameters across all experimental groups.

### RNA extraction and qRT-PCR assay

Total RNA was extracted using TRIzol reagent (Life Technology, USA) according to the manufacturer's protocol and resuspended in 20 µl RNase-free water. The RNA concentration was determined using an ultra-micro spectrophotometer (Quawell, USA). The cDNAs were synthesized using Prime Script RT Master Mix kit (Takara, Japan), and their mRNAs expression was quantitatively analyzed by the SYBR Prime Script RT-PCR kit (Takara, Japan). Quantitative PCR was performed using the CFX96 Touch real-time PCR detection system (Bio-Rad, USA) with the following thermocycling conditions: Initial denaturation at 95˚C for 30 seconds, followed by 40 cycles of denaturation at 95˚C for 5 seconds and annealing/extension at 60˚C for 30 seconds. The β-actin mRNA was used as normalization control, and the primer sequences for qPCR were presented in **Table S1.**

### Western blot analysis

Briefly, the cells were washed with cold PBS and subsequently lysed with RIPA lysis buffer (GenView, USA) on ice for 10 minutes. The lysates were centrifuged at 18,000 g at 4˚C for 15 min. Protein concentrations were determined with the BCA protein assay kit (Sigma-Aldrich, USA). The samples were denatured by heating in SDS running buffer at 100˚C for 10 minutes. The equal amounts of denatured protein samples were separated on 6% to 10% (w/v) sodium dodecyl sulfate-polyacrylamide gel (SDS-PAGE), followed by transfer onto polyvinylidene fluoride (PVDF) membranes (Bio-Rad, USA). The membranes were blocked with 5% BSA for 1 h at room temperature. The membranes were incubated with the primary antibodies (1:2,000 dilution) overnight at 4˚C. And then, the membranes were incubated with horseradish peroxidase-linked secondary antibodies (1:10,000 dilution) for 1h at room temperature. After being washed with TBST, the membranes were exposed to the ChemiDoc XRS System (Bio-Rad, USA) to detect the expressions of the target proteins, which were enhanced by using the ECL Kit (GenView, USA) and quantified with Image Lab software (Bio-Rad, USA), with normalization to β-actin levels.

### Statistical analysis

The results were expressed as means and standard deviations (SD). Analysis of variance (ANOVA) was employed to assess the significance of differences, with multiple comparisons corrected using Dunnett's Method. A significance level of P < 0.05 was considered statistically significant, while a significance level of P < 0.01 was considered extremely statistically significant. Statistical analyses were performed with the SPSS statistical software package, and biological replicates were utilized in all experiments.

## Results

### Platelet-CTCs interactions promote the secretion of TGF-β and induce the activation of pSmad expression

Platelet-CTCs interactions play a crucial role in enabling CTCs to evade immune cell attack while enhancing their survival and transendothelial migration. This process is facilitated by the release of TGF-β. Here, we found that platelet-HT29 cells interactions promoted platelet secretion of TGF-β and induced cancer cells expression of phosphorylated Smad (pSmad). As illustrated in **Supplementary Figure 1A**, the interaction between platelets and CTCs resulted in a substantial increase in TGF-β secretion—nearly 1000 pg/mL—regardless of the presence of adenosine diphosphate (ADP), a platelet activator. In contrast, the TGF-β levels in the control group, which consisted of platelet-poor plasma (PPP), were approximately 300 pg/mL. Additionally, our results demonstrate that platelets significantly upregulate pSmad expression in HT29 cells compared to the control group (**Supplementary Figure 1B**). These findings suggest that platelets enhance the EMT and metastasis of cancer cells through the secretion of TGF-β, which activates pSmad expression.

### TGF-β promotes the invasiveness and metastatic potential of colorectal cancer cells by inducing EMT

**Figure 1:**
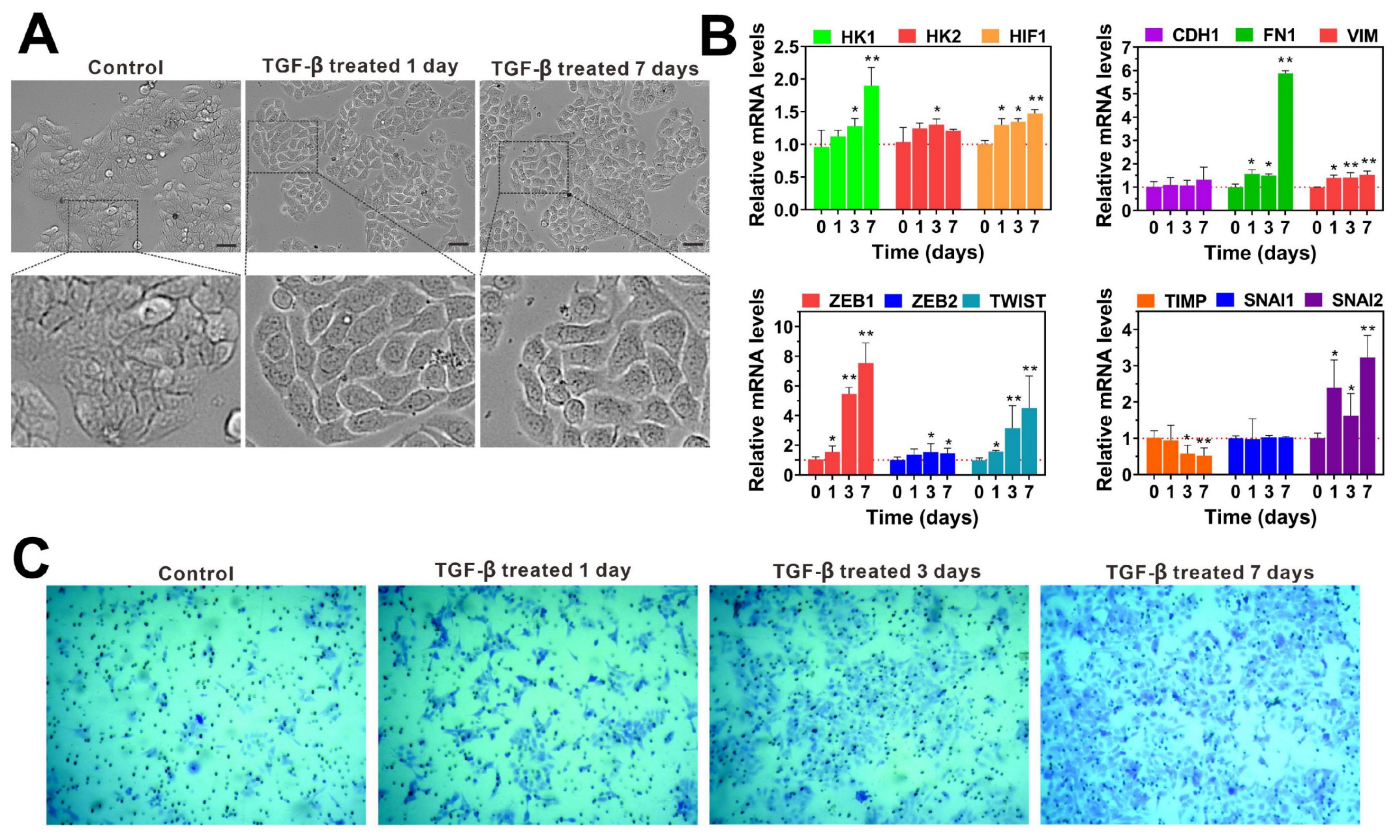
TGF-β enhances the invasion of colorectal cancer cells by inducing epithelial-to-mesenchymal transition (EMT). **(A)** The cell morphology of HT29 cells was observed under a microscope (scale bar 20 µm) after treatment with TGF-β for different time periods (0, 1, and 7 days). **(B)** The qRT-PCR analysis showed that TGF-β significantly upregulated the expression of EMT-related transcription factors in HCT116 cells. mRNA expressions were normalized to β-actin, and results are calculated as ratio of the control group (without TGF-β treatment). **(C)** The cell invasion ability was determined by transwell assay in HCT116 cells treated with TGF-β for different durations (0, 1, 3, and 7 days). Data are the mean ± SD (n=3). *p < 0.05, **p < 0.01 compared to the control group (without TGF-β treatment).

The EMT is a dynamic process involved in cancer metastasis, characterized by the acquisition of a mesenchymal phenotype and a reduction in intercellular adhesion, which enhances cellular motility 30, 31. Numerous studies have established that TGF-β is a key regulator of EMT, and models of TGF-β-induced EMT have been applied across various cancer types 32, 33. In our experiments, we observed that TGF-β treatment induces EMT in colon cancer cell lines (HT29, LoVo, HCT116) compared to untreated controls. Detailed microscopic analysis of TGF-β-treated cells, as shown in **Figure 1A and Supplementary Figure 2A**, revealed that after 24 hours, these cells began to exhibit mesenchymal characteristics. After seven days of exposure to TGF-β, we noted the emergence of spindle-shaped cells with increased pseudopodia formation and larger intercellular spaces.

To confirm TGF-β's role in EMT activation, we examined the expression of EMT-related mRNA in HT29, HCT116 and LoVo cells using qRT-PCR for various time periods. The results are shown in **Figure 1B**, treatment with TGF-β led to a significant time-dependent upregulation of HK1, HK2, HIF1, FN1, VIM, ZEB1, SNAIL2, and TWIST mRNA levels of HCT116 cells compared to the control group. Similarly, when exposure of HT29 and LoVo cells to TGF-β for 7 days resulted in a remarkable increase in HK1, HK2, HIF1, FN1, VIM, ZEB1, ZEB2 and SNAIL2 mRNA levels (**Supplementary Figure 2B and 2C**). These findings suggest that TGF-β promotes EMT by upregulating mRNA levels of key EMT-inducing factors. Additionally, we examined the effect of TGF-β on aggressive behaviors of HT29 and HCT116 cells *in vitro*. The transwell assay showed that TGF-β obviously increased the invasion of HCT116 and HT29 cells in a time-dependent manner when compared to untreated controls (**Figure 1C** and **Supplementary Figure 3**). These results highlight TGF-β's role in driving cancer progression through the induction of EMT, which enhances cellular invasiveness and the potential for metastasis.

**Figure 2:**
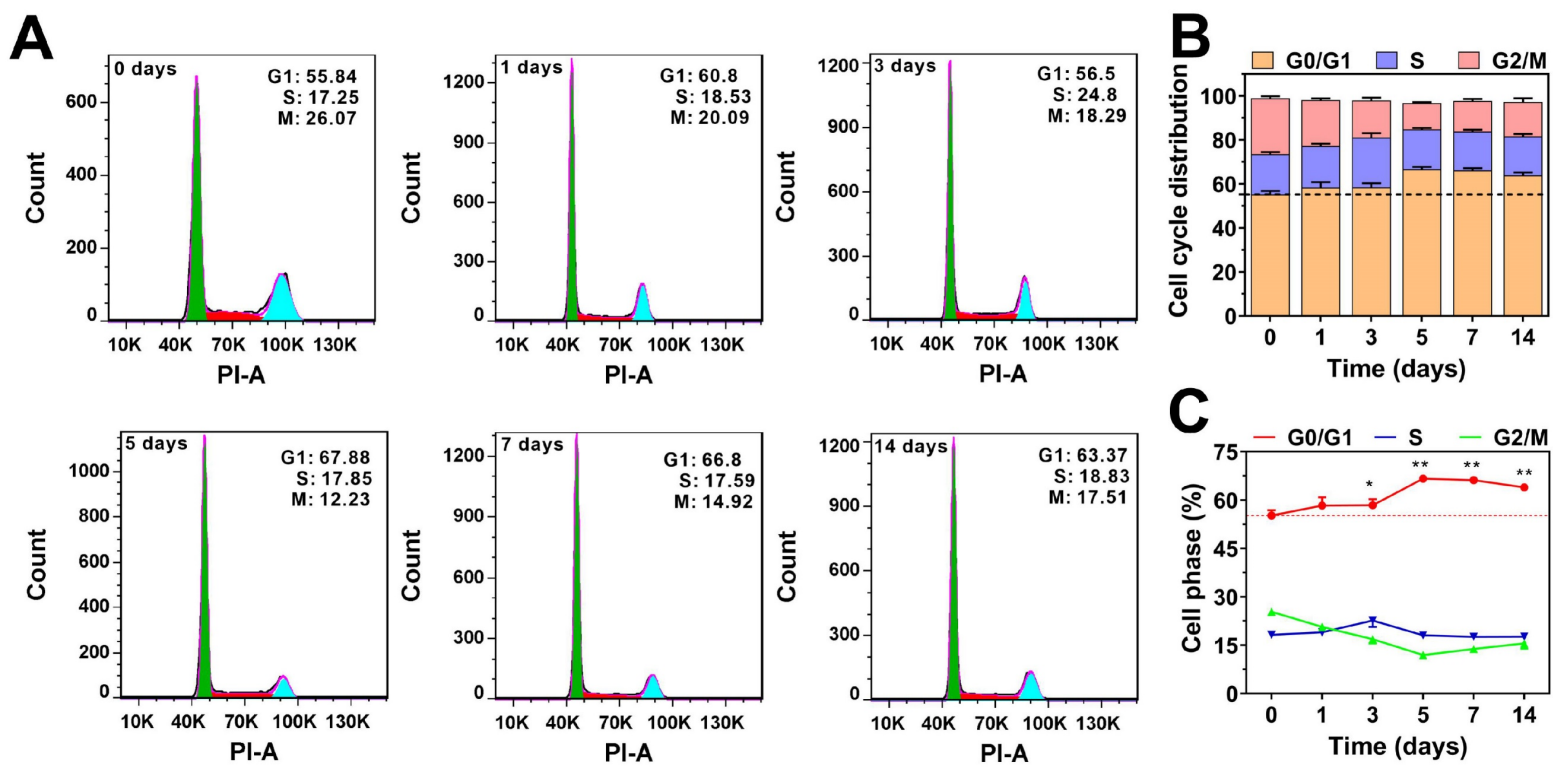
Effect of TGF-β on cell cycle distribution. **(A)** HCT116 cells were treated with TGF-β (10 ng/mL) for 0, 1, 3, 5, 7, and 14 days. The cell cycle was determined by flow cytometry, and the distribution of cell cycle was analyzed using FlowJo software. **(B)** Quantitative analysis of the effect of TGF-β on the cell cycle distribution in HCT116 cells. **(C)** The percentage of cells in G0/G1, S, and G2/M phases as a function of time in HCT116 cells. Data are the mean ± SD (n=3). *p < 0.05, **p < 0.01 compared to the control group (0 days).

We also analyzed the effect of TGF-β on the cell cycle distribution of HCT116 cells. As shown in **Figure 2**, TGF-β treatment resulted in a slight increase in the proportion of cells in the G0/G1 phase, while the proportion of cells in the mitotic phases (S and G2/M) decreased slightly. After prolonged TGF-β treatment (more than five days), the percentage of cells in the G0/G1 phase stabilized at around 65%, showing little variation over time. These findings indicate that TGF-β modulates the cell cycle to adapt to the cancer microenvironment, maintaining a balance between proliferation and migration. Collectively, these results strongly support the notion that TGF-β is crucial in regulating the cell cycle and acts as a driving force in cancer metastasis.

### TGF-β enhances glucose consumption and ATP synthesis through the activation of AKT signaling pathways in cancer cells

**Figure 3:**
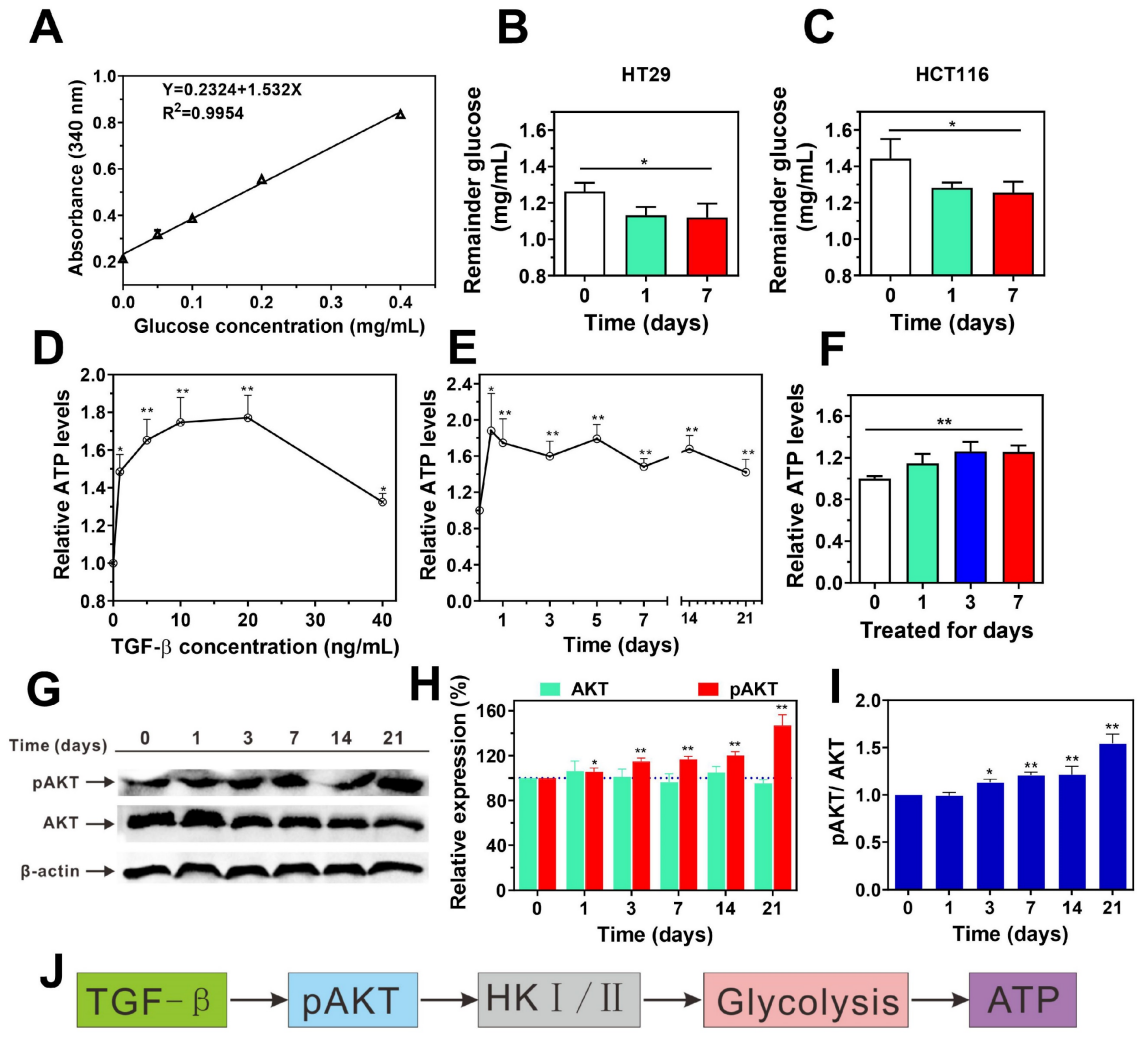
TGF-β promotes glucose metabolism and ATP synthesis by activating the AKT signaling pathway. **(A)** The standard curve of glucose concentration. **(B and C)** the remainder glucose after TGF-β treatment in HT29 (B) and HCT116 (C) cells was determined using Glucose (HK) Assay Kit. Data are the mean ± SD (n=3). *p < 0.05 compared to the control group (0 days). **(D)** The levels of ATP were determined in HCT116 cells treated with TGF-β (0, 1, 5, 10, 20, 40 ng/mL) for 24 h using ATP Assay Kit. **(E)** The levels of ATP were determined in HCT116 cells treated with 10 ng/mL TGF-β for different time periods (0, 0.5, 1, 3, 5, 7 14, and 21 days) using ATP Assay Kit. **(F)** The ATP levels was determined using ATP assay kit in HT29 cells treated with 10 ng/mL TGF-β for 0, 1, 3, and 7 days. The relative ATP levels were calculated as a ratio of control group (0 days). **(G-I)** HCT116 cells were treated with TGF-β (10 ng/mL) for 0, 1, 3, 7, 14, and 21 days. The expression levels of p-AKT and AKT were determined by western blot, β-actin was used as the loading control. Band intensity was quantified using Image Lab analysis software and calculated as percentage (H) or ratio (I) of control group (0 days). **(J)** Schematic diagram illustrates the mechanism of TGF-β-induced glucose metabolism and ATP synthesis in HCT116 cells. Data are the mean ± SD (n=3). *p < 0.05, **p < 0.01 compared to the control group (0 days).

Metastasis requires enhanced invasiveness from cancer cells, which significantly raises their energy demands compared to normal cells. This phenomenon, known as the "Warburg effect," was first described by Otto Warburg in the 1920s and has since become a well-established concept in cancer biology 34. Building on this foundation, we investigated how TGF-β influences intracellular glucose consumption and ATP production in cancer cells. Using a glucose assay kit, we measured glucose uptake in HT29 and HCT116 cells after TGF-β treatment. As shown in **Figure 3A-C**, TGF-β significantly enhanced glucose consumption in both cell lines. Interestingly, a 24-hour exposure of HCT116 cells to TGF-β led to a concentration-dependent rise in ATP levels, peaking at 10 ng/mL. Beyond this concentration, ATP levels began to decline, suggesting possible cytotoxic effects at higher TGF-β levels (**Figure 3D**). Consequently, we selected 10 ng/mL for subsequent experiments. At this concentration, TGF-β significantly raised ATP levels in both HCT116 and HT29 cells after 12 hours, sustaining high ATP synthesis over time (**Figure 3E-F**). These observations indicate that TGF-β may induce metabolic reprogramming in cancer cells, resulting in enhanced glucose consumption and ATP production.

Cancer cells predominantly generate ATP via aerobic glycolysis within mitochondria, which supports various cellular activities 35. Hexokinase (HK) is the first enzyme in glycolysis, catalyzing glucose to glucose-6-phosphate (G6P) 36, thus playing a crucial role in regulating glucose phosphorylation 37. There are four subtypes of HK, namely HK1, HK2, HK3 and HK4, among which HK1 and HK2 play a role as drivers of cancer cell glycolysis 36. Recent studies indicate that AKT plays an important role in regulating the expression of HK1 and HK2 38. In current study, we found that the mRNA transcriptional levels of HK1 and HK2 in HT29, LoVo, and HCT116 cells after TGF-β treatment were significantly higher than those in normal cells without TGF-β treatment (**Figure 1B** and **Supplementary Figure 2B-C**). Furthermore, the administration of TGF-β resulted in an elevation in the ratios of p-AKT/AKT in HCT116 cells compared to those observed in the control group (**Figure 3G-I**). These findings suggest that TGF-β upregulates HK1 and HK2 expression via AKT pathway activation, which may drive enhanced glycolysis and ATP synthesis in cancer cells (**Figure 3J**).

### TGF-β-induced highly metastatic HCT116 cells exhibit increased bioenergetic potential and environmental adaptability

Our previous study demonstrated that TGF-β induces EMT in colon cancer cells, increasing their metastatic potential. Additionally, TGF-β enhances metabolic activity and modulates proliferation rates, which may improve the environmental adaptability and survival of TGF-β-induced metastatic cancer cells. To explore these effects, we compared the stress responses of TGF-β-induced highly metastatic HCT116 cells (H-HCT116) and untreated HCT116 cells under energy-limited conditions by inhibiting oxidative phosphorylation (OXPHOS) with oligomycin A.

**Figure 4:**
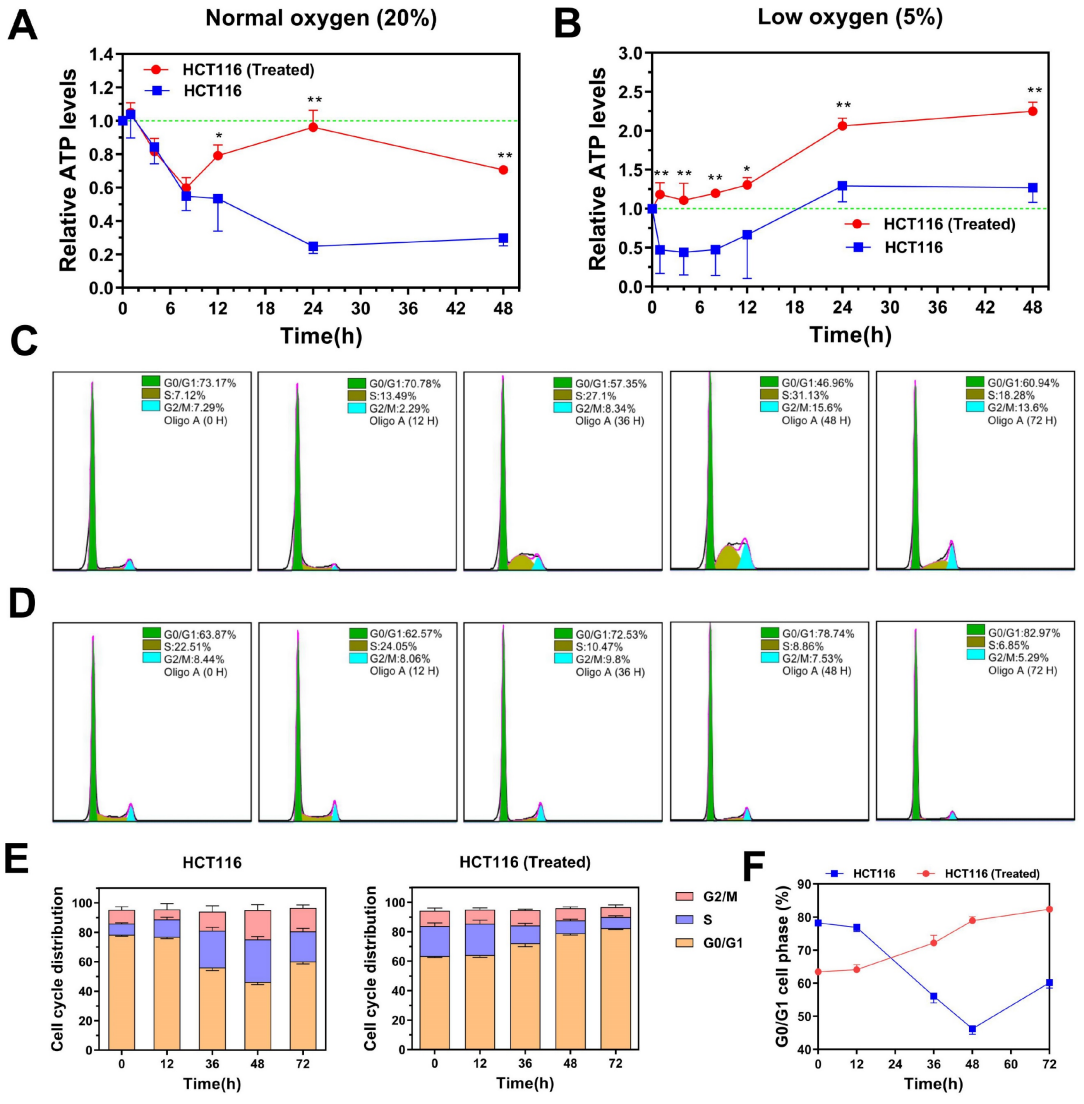
TGF-β-induced highly metastatic HCT116 (H-HCT116) cells exhibit increased bioenergetic potential and cell cycle distribution adaptability. **(A)** H-HCT116 was obtained by TGF-β (10 ng/mL) treatment for 7 days. In normal oxygen (20%) culture environment, the internal ATP levels were determined in HCT116 and H-HCT116 cells treated with oligomycin A (1 µM) for 0-48 h.** (B)** In low oxygen (5%) culture environment, the internal ATP levels were determined in HCT116 and H-HCT116 cells treated with oligomycin A (1 µM) for 0-48 h. Relative ATP levels in (A) and (B) were calculated as a ratio of control group (0 h). Data are the mean ± SD (n=3). *p < 0.05, **p < 0.01 compared to the HCT116 cells.** (C and D)** The cell cycle distribution of HCT116 cells (C) and H-HCT116 cells (D) was determined using flow cytometry after treatment with oligomycin A (1 µM) for 0, 12, 36, 48, and 72 h, respectively. **(E)** Quantitative analysis of the adjustment by oligomycin A on the cell cycle distribution of HCT116 cells and H-HCT116 cells. **(F)** Percentage of cells in G0/G1 phase as a function of time in HCT116 cells and H-HCT116 cells. Data are the mean ± SD (n=3). HCT116 (Treated) indicates H-HCT116 cells.

Under normal oxygen levels (20%), untreated HCT116 cells exhibited a rapid drop in ATP levels after oligomycin A treatment (1 μM), with ATP levels declining to 20% of the baseline within 24 hours (**Figure 4A**). In contrast, H-HCT116 cells showed only a gradual reduction in ATP levels within the first six hours of oligomycin A treatment, maintaining approximately 60% of baseline levels. Notably, after this initial drop, ATP levels in H-HCT116 cells began to increase, nearly returning to baseline after 24 hours. These findings suggest that H-HCT116 cells may rapidly activate alternative energy pathways, such as glycolysis, to compensate for energy deficits when OXPHOS is inhibited by oligomycin A.

In hypoxic conditions (5% oxygen), ATP levels in untreated HCT116 cells decreased to 50% of baseline following oligomycin A exposure. After 12 hours, ATP levels in these cells began to recover, eventually returning to normal (**Figure 4B**). In H-HCT116 cells, however, ATP levels did not decline with oligomycin A treatment but instead showed a steady increase, reaching nearly double the baseline by 24 hours. These findings suggest that in low-oxygen conditions, HCT116 cells primarily rely on glycolysis due to reduced OXPHOS, limiting oligomycin A's effect. H-HCT116 cells, however, demonstrate an enhanced capacity to upregulate alternative energy pathways, leading to a substantial boost in energy production. These results suggest that TGF-β-induced H-HCT116 cells possess increased adaptability to harsh microenvironments and can dynamically adjust metabolic processes in response to energy limitations.

We further examined the effect of OXPHOS inhibition by oligomycin A on cell cycle distribution in H-HCT116 and untreated HCT116 cells. While oligomycin A significantly reduced ATP levels in HCT116 cells, it led to an increase in the proportion of cells in the division phase (**Figure 4C**). In contrast, prolonged exposure to oligomycin A in H-HCT116 cells resulted in a gradual increase in the G0/G1 phase population, with a corresponding decrease in cells undergoing division (**Figure 4D-F**). These findings indicate that, under energy constraints, H-HCT116 cells may reduce proliferation rates to conserve energy for survival, effectively maintaining viability. Collectively, these observations suggest that H-HCT116 cells have an enhanced ability to regulate the balance between survival and proliferation in response to environmental stressors.

### TGF-β-induced highly metastatic HCT116 cells exhibit activated hexokinase expression and inhibited cyclin expression in the presence of OXPHOS inhibition

**Figure 5:**
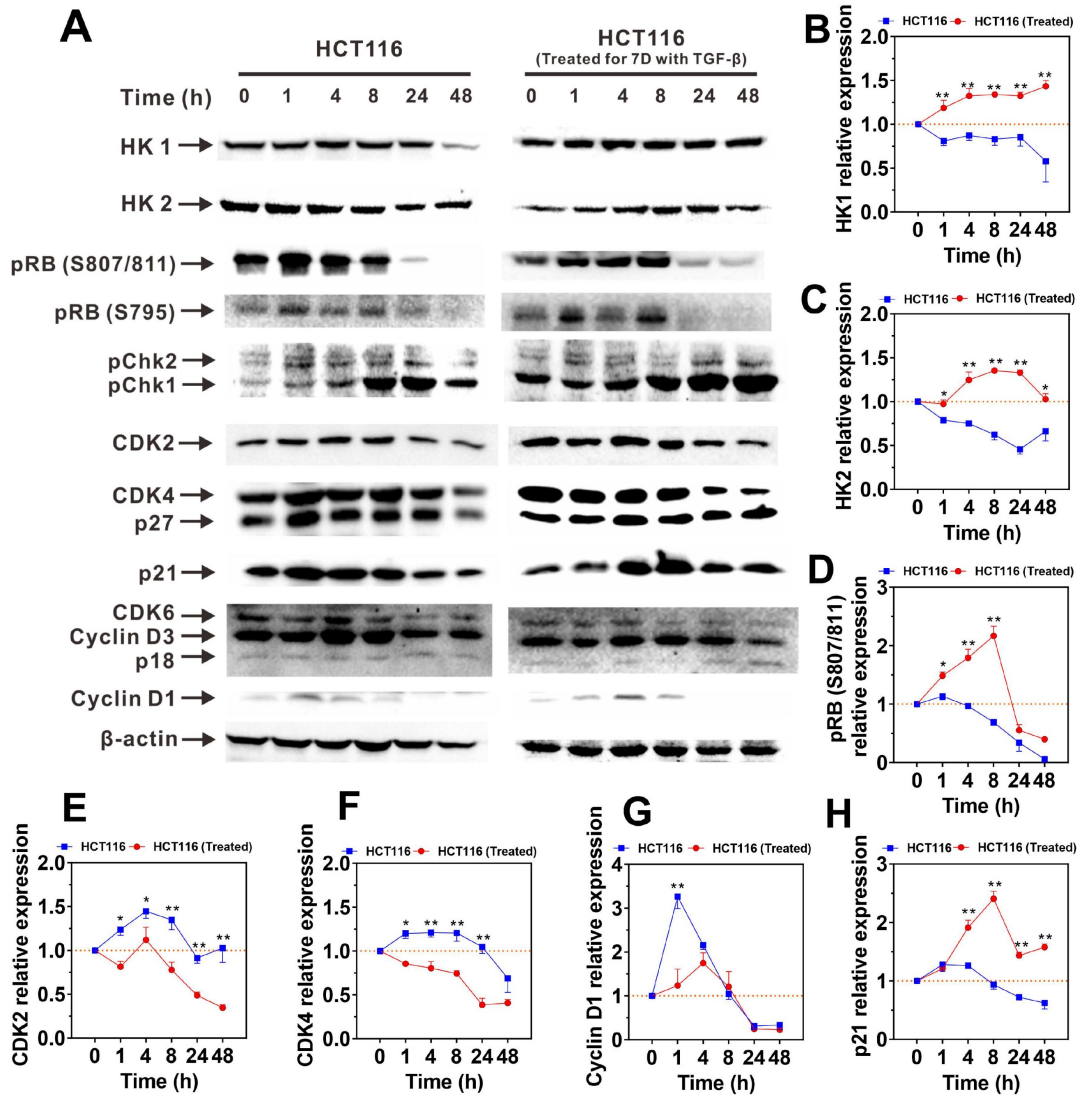
The expression of HK and cell cycle regulatory proteins in HCT116 and TGF-β-induced highly metastatic HCT116 (H-HCT116) cells with inhibition of oxidative phosphorylation (OXPHOS). **(A)** H-HCT116 was obtained by TGF-β (10 ng/mL) treatment for 7 days. HCT116 cells and H-HCT116 cells were treated with oligomycin A (1 μM) for 0, 1, 4, 8, 24, and 48 h. The expression levels of HK and cell cycle regulatory proteins were determined by western blot, β-actin was used as the loading control. **(B-H)** Band intensity were quantified by using Image Lab analysis software and calculated as ratio of control group (0 h). HCT116 (Treated) indicates H-HCT116 cells. Data are the mean ± SD (n=3). *p < 0.05, **p < 0.01 compared to the HCT116 cells.

To clarify the mechanisms that enhance the adaptability and metabolic capacity of H-HCT116 in energy-limited environments, we examined changes in the protein levels of hexokinase isoforms HK1 and HK2 following oligomycin A treatment. As shown in **Figure 5A**, untreated HCT116 cells displayed a reduction in HK1 and HK2 expression over time with oligomycin A exposure. Conversely, H-HCT116 cells exhibited a gradual increase in both proteins initially, with HK2 levels declining only after 48 hours (**Figure 5B-C**). These protein expression patterns align with previous observations of TGF-β-induced upregulation of HK mRNA (**Figure 1B** and **Supplementary Figure 2**) and intracellular ATP maintenance (**Figure 4A-B**). These findings suggest that TGF-β-induced H-HCT116 cells can upregulate HK expression and glycolytic metabolism, thereby compensating for the reduced energy production from impaired OXPHOS.

We further investigated the expression changes of cell cycle switch proteins and regulatory proteins following treatment with oligomycin A. The results revealed that the expression patterns of cell cycle switch proteins pRB (S795), pChk1 and pChk2 in both HCT116 and H-HCT116 cells were similar upon exposure to oligomycin A (**Supplementary Figure 4A-C**). The expression of pRB (S807/811) initially increased in H-HCT116 cells, followed by a subsequent decrease, aligning with the trend observed in HCT116 cells (**Figure 5D**). Conversely, the expression levels of cell cycle regulatory proteins CDK2, CDK4 and Cyclin D1 increased in HCT116 cells but decreased in H-HCT116 cells after oligomycin A treatment (**Figure 5E-G**). The expression levels of CDK6, Cyclin D3 and P27 in both HCT116 and H-HCT116 cells remained consistent upon exposure to oligomycin A (**Supplementary Figure 4D-F**). Additionally, while P21 expressions decreased in untreated HCT116 cells, they were upregulated in H-HCT116 cells (**Figure 5H**). It is known that phosphorylated retinoblastoma protein (pRB) promotes cell cycle progression from the G0 phase 39, while the phosphorylation of Chk (pChk) promotes the closure of G2/M phase 40, 41. The increased expression of CDK2, CDK4, CDK6, Cyclin D1 and Cyclin D3 promotes cell division, while the increased expression of p18, p21, p27 inhibits cell division 42-44. Our results indicate that TGF-β-induced H-HCT116 cells modulate cell division rates by suppressing CDK2, CDK4, and Cyclin D1 expression while enhancing p21 expression under conditions of limited energy (such as OXPHOS inhibition). These adaptations allow H-HCT116 cells to conserve energy, indicating an advanced survival mechanism in resource-scarce conditions.

### TGF-β-induced highly metastatic HCT116 cells exhibit enhanced cellular deformation and motility in the presence of OXPHOS inhibition

**Figure 6:**
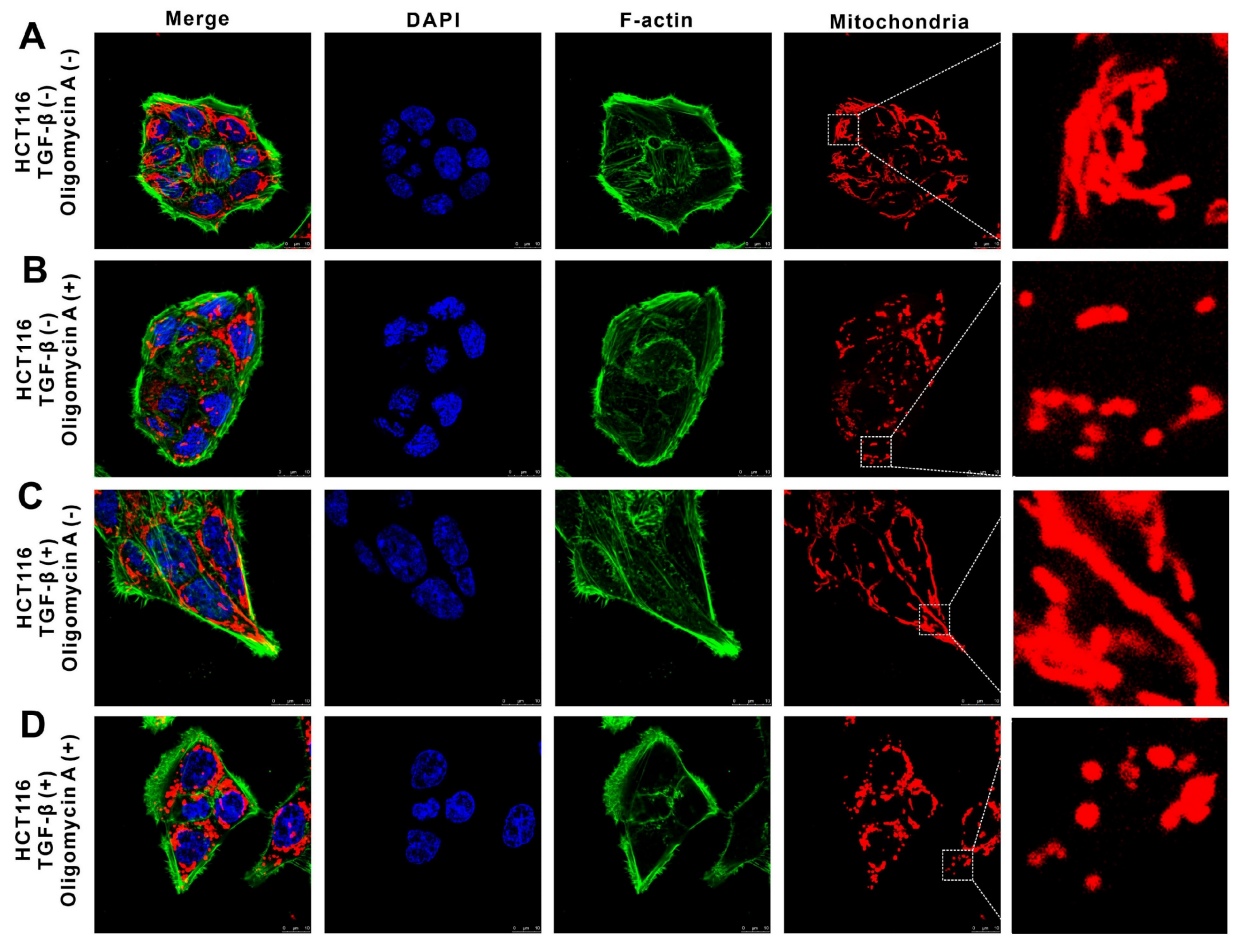
The effect of oligomycin A on deformation and motility in HCT116 and TGF-β-induced highly metastatic HCT116 (H-HCT116) cells. H-HCT116 was obtained by TGF-β (10 ng/mL) treatment for 7 days. The distribution of mitochondria and Factin was observed in HCT116 and H-HCT116 cells using a confocal laser scanning microscope. Mitochondrial were stained with Mito Tracker Red CMXRos (red), F-actin were stained with phallotoxins-Alexa Fluor 488 (green), and the cell nucleus were stained with DAPI (blue). The scale bar represents 10 µm. H-HCT116 was obtained by TGF-β (10 ng/mL) treatment for 7 days. **(A)** represent normal HCT116 cells, **(B)** represent HCT116 cells treated with oligomycin A for 24 h,** (C)** represent H-HCT116 cells, **(D)** represent H-HCT116 cells treated with oligomycin A for 24 h.

Using immunofluorescence staining and confocal laser scanning microscopy, we visualized the distribution of mitochondria and cytoskeleton components in both HCT116 and TGF-β-induced metastatic H-HCT116 cells after oligomycin A treatment. In untreated HCT116 cells, F-actin (green, Alexa Fluor 488-labeled) displayed a regular, even distribution, forming an oval or spherical shape with a smooth surface. Mitochondria (red) showed a linear arrangement, indicating organized mitochondrial metabolism (**Figure 6A**). Upon oligomycin A treatment, the F-actin distribution in HCT116 cells remained largely unchanged, while the mitochondrial arrangement shifted from linear to punctate, reflecting a reduction in mitochondrial activity (**Figure 6B**). In contrast, H-HCT116 cells demonstrated a fusiform or fibrous shape with pseudopodia extensions and a denser distribution of F-actin. Their mitochondria were arranged in linear structures within the cell (**Figure 6C**). After 24 hours of oligomycin A exposure, H-HCT116 cells adopted a more elongated shape, with densely concentrated F-actin in the pseudopodia, and a subset of mitochondria transitioned from linear to granular structures (**Figure 6D**). These observations indicate that H-HCT116 cells exhibit a mesenchymal morphology, with abundant F-actin density in pseudopodia, which supports their increased motility and invasive capacity. While most mitochondria in untreated HCT116 cells displayed low metabolic activity (small, grain-like shapes), H-HCT116 cells retained active mitochondrial regions despite oligomycin A exposure, highlighting their enhanced resilience to the inhibitory effects on oxidative phosphorylation. These findings suggest that TGF-β-induced highly metastatic HCT116 cells can maintain mitochondrial activity and cytoskeletal dynamics under metabolic stress, facilitating their motility and invasiveness.

## Discussion

**Figure 7:**
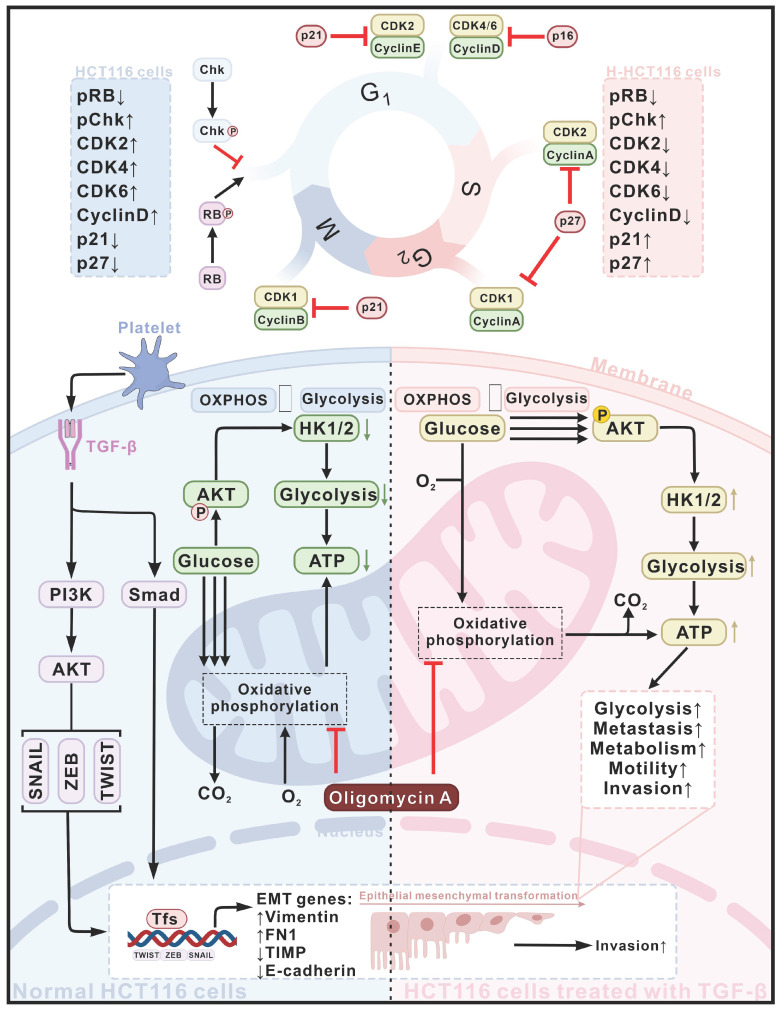
Schematic diagram of the mechanism of TGF-β activates cancer metastasis potential by inducing metabolic reprogramming and bioenergetic adaptation. Platelet-CTCs interactions lead to the secretion of TGF-β, which triggers the activation of PI3K-AKT and Smad signaling pathways, thereby inducing EMT in HCT116 cells (depicted in the blue section), ultimately transforming them into highly metastatic HCT116 (H-HCT116) cells (depicted in the pink section). On the one hand, TGF-β stimulates the expression of HK1 and HK2 in CTCs by activating the AKT signaling pathway, inducing metabolic reprogramming in them. H-HCT116 cells can compensate for impaired oxidative phosphorylation (OXPHOS) energy supply caused by oligomycin A treatment by upregulating HK expression and glycolytic metabolism. On the other hand, H-HCT116 cells regulate the rate of cell division by downregulating the expression of CDK2, CDK4, and Cyclin D1 proteins while upregulating the expression of p21. This reduces cell proliferation to conserve energy for survival when OXPHOS is inhibited by oligomycin A.

The present studies have observed that interactions between platelets and CTCs promote TGF-β secretion, which subsequently induces pSmad expression in CTCs. TGF-β also activates the AKT signaling pathway in CTCs, driving the expression of HK1 and HK2 and triggering EMT. Together, these effects enhance metabolic reprogramming, modulate cell cycle progression, and increase the metastatic potential of CTCs. Notably, TGF-β-induced highly metastatic HCT116 (H-HCT116) cells can adapt to compromised OXPHOS by upregulating HK expression and shifting toward glycolytic metabolism. Concurrently, these cells limit proliferation rates through the downregulation of CDK2, CDK4, and Cyclin D1, while upregulating p21 to conserve energy for survival. These observations underscore that TGF-β, secreted through CTCs-platelet interactions, enhances cancer metastasis by driving metabolic reprogramming and bioenergetic adaptation (**Figure 7**).

EMT involves a loss of epithelial characteristics and a shift toward mesenchymal phenotypes 10, 11, 45, thereby significantly increasing cell migration and invasion potential undergo metabolic shifts, particularly with glycolysis being markedly activated (**Figure 3**). Compared to oxidative phosphorylation, glycolysis can quickly generate substantial energy, supporting the survival and proliferation of cancer cells during metastasis. Under conditions of energy stress, cancer cells further increase glycolytic activity to meet energy demands (**Figure 4A-B**). Research indicates that increased glycolysis can reinforce EMT, creating a positive feedback loop 46. Both EMT and glycolysis are by signaling pathways, including PI3K/AKT and HIF-1α, whose activation facilitates both processes (**Figures 1 and Figure 3**).

While the study focuses on colon cancer, the observed mechanisms—platelet-CTCs interactions promoting TGF-β release and subsequent pSmad activation—may have broader implications across various cancer types. Many cancers rely on similar pathways to enhance metastasis, evade immune responses, and adapt to the blood microenvironment. Thus, elucidating these interactions in colon cancer could potentially offer insights into metastatic processes in other solid tumors, such as breast, lung, and pancreatic cancers, where TGF-β signaling and platelet-tumor cell interactions are also critical.

Metabolic reprogramming is the adaptive modification of cancer cell metabolism, marked by a shift from OXPHOS to glycolysis, enabling rapid energy production 47, 48. As EMT enhances cell migration and additional energy demands met by metabolic reprogramming, especially via elevated glycolytic activity. Our findings demonstrate that TGF-β activates Smad and PI3K/AKT pathways, simultaneously driving EMT and metabolic reprogramming (**Figure 1, Supplementary Figures 1-3**). EMT, in turn, promotes metabolic reprogramming by altering intracellular metabolic conditions, while the metabolic shift fuels EMT by supplying energy and biosynthetic precursors. In energy-deficient states, TGF-β-induced highly metastatic cancer cells undergo adaptive metabolic changes, such as cell cycle arrest, to prioritize energy for survival and migration (**Figure 4-6**). The mutual reinforcement between EMT and metabolic reprogramming establishes a complex regulatory network with critical implications for tumor growth, proliferation, and metastasis.

However, this study has certain limitations, particularly its specificity to the cell lines used and the lack of *in vivo* validation. Future research will address these limitations to provide a more comprehensive foundation for the study's conclusions and to strengthen its potential for clinical translation. By expanding on these aspects, we aim to facilitate the transition of these findings from the laboratory to clinical applications, ultimately contributing to the development of new interventions to reduce cancer metastasis.

## Conclusion

In conclusion, the present studies demonstrated that interactions between platelets and CTCs drive TGF-β secretion, which in turn promotes metabolic reprogramming in cancer cells. This process enhances glycolytic activity and increases cellular invasiveness by activating EMT. Additionally, suppression of OXPHOS in TGF-β-induced highly metastatic HCT116 cells further amplifies glycolysis, allowing these cells to optimize energy utilization by reducing cell division rates and increasing their migratory capacity. Collectively, these results suggest that targeting glycolysis and metabolic reprogramming pathways could be an effective strategy to prevent cancer metastasis.

## Supplementary Material

Supplementary figures and table.
