# Supplementary material for: TGF-β secreted by cancer cells-platelets interaction activates cancer metastasis potential by inducing metabolic reprogramming and bioenergetic adaptation
Source: J Cancer. 2025 Jan 13;16(4):1310–23. doi: 10.7150/jca.103757 (PMC11786022; doi:10.7150/jca.103757)
Supplement: Supplementary file 1 — Supplementary figures and table. [file jcav16p1310s1.pdf]

Supplementary tables

Supplementary Table 1. Primer sequences used for reverse transcription-quantitative polymerase chain reaction analyses of human colorectal cancer cell lines HT29, HCT116, and LoVo.

Gene	Forward primer sequence (5'-3')	Reverse primer sequence (5'-3')
HK1	AATGCTGGGAAACAAAGGT	AGAGGAATCCCTTCTTGGG
HK2	CCTCGGTTTCCCAACTCTGC	GCTCCAAGCCCTTTCTCCAT
ZEB1	ATCCTGGGGCCTGAAGCTCAGG	TGGTGTGCCCTGCCTCTGGT
ZEB2	ACGGTATTGCCAACCCTCTG	CTCCCTTATTTCATCTTCCTCTTC
SNAIL1	GCTGCTACAAGGCCATGTCCGG	CTTGGTGCTTGTGGAGCAGGGAC
SNAIL2	GATGCCGCGCTCCTTCCTGG	TGGAGCAGCGGTAGTCCACAC
FN1	TGCAAGGCCTCAGACCGGGT	GCGCTCAGGCTTGTGGGTGT
VIM	TTCCAAGCCTGACCTCACGGCTG	TTCCGGTTGGCAGCCTCAGAGA
CDH1	CCGAGGACTTTGGCGTGGGC	TCCCTGTCCAGCTCAGCCCG
HIF1	CGTTCCTTCGATCAGTTGTC	TCAGTGGTGGCAGTGGTAGT
CD133	CAGAGTACAACGCCAAACCA	AAATCACGATGAGGGTCAGC
TWIST	TCCGCGTCCCACTAGCAGGC	CGCCCCACGCCCTGTTTCTT
MMP2	GGCATTCAGGAGCTCTATGG	GAGCGATGCCATCAAATACA
TIMP	GTAGTGATCAGGGCCAAAGC	CAGGCCCTTTGAACATCTTT
β-actin	AACTGGGACGACATGGAGAAAA	GGATAGCACAGCCTGGATAGCA

HK, Hexokinase; ZEB, Zinc-finger E-box-binding homeobox; SNAIL, snail family transcriptional repressor; FN1, Fibronectin 1; VIM, Vimentin; CDH1, Epithelial-cadherin; HIF1, Hypoxia-inducible factor 1α.

Supplementary Figures

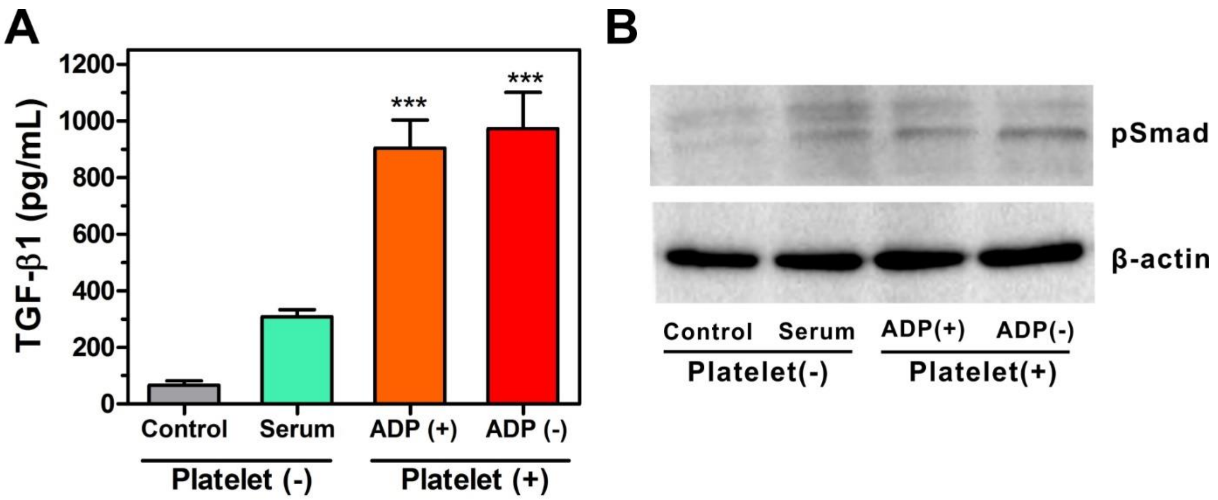

Supplementary Figure 1. Platelet-CTCs interactions promote the secretion of TGF-β and induce the activation of pSmad expression. (A) Concentration of TGF-β1 in conditioned medium from HT29 cells treated with buffer or platelets for 4 h. **(B)** pSmad expression of HT29 cells treated with buffer or platelets for 24 h.

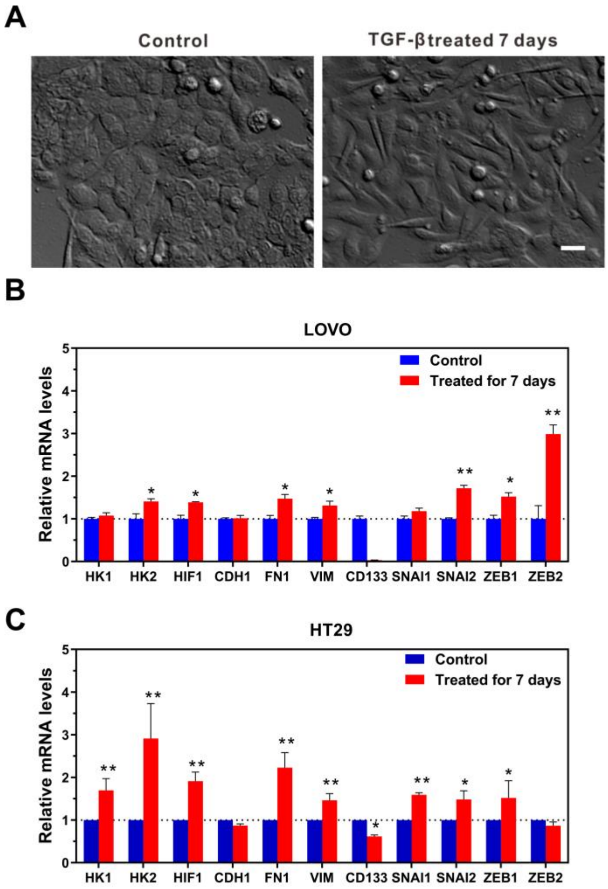

Supplementary Figure 2. TGF-β induces epithelial-to-mesenchymal transition (EMT) in colorectal cancer LOVO and HT29 cells. (A) The cell morphology of LOVO cells was observed under a microscope after treatment with TGF-β for 0 and 7 days). **(B and C)** The qRT-PCR analysis showed that TGF-β significantly upregulated the expression of EMT-related transcription factors in LOVO **(B)** and HT29 **(C)** cells. mRNA expressions were normalized to β-actin, and results are calculated as percentage of the control group (without TGF-β treatment). Data are the mean ± SD (n=3). *$p < 0.05$, **$p < 0.01$ compared to the control group (without TGF-β treatment).

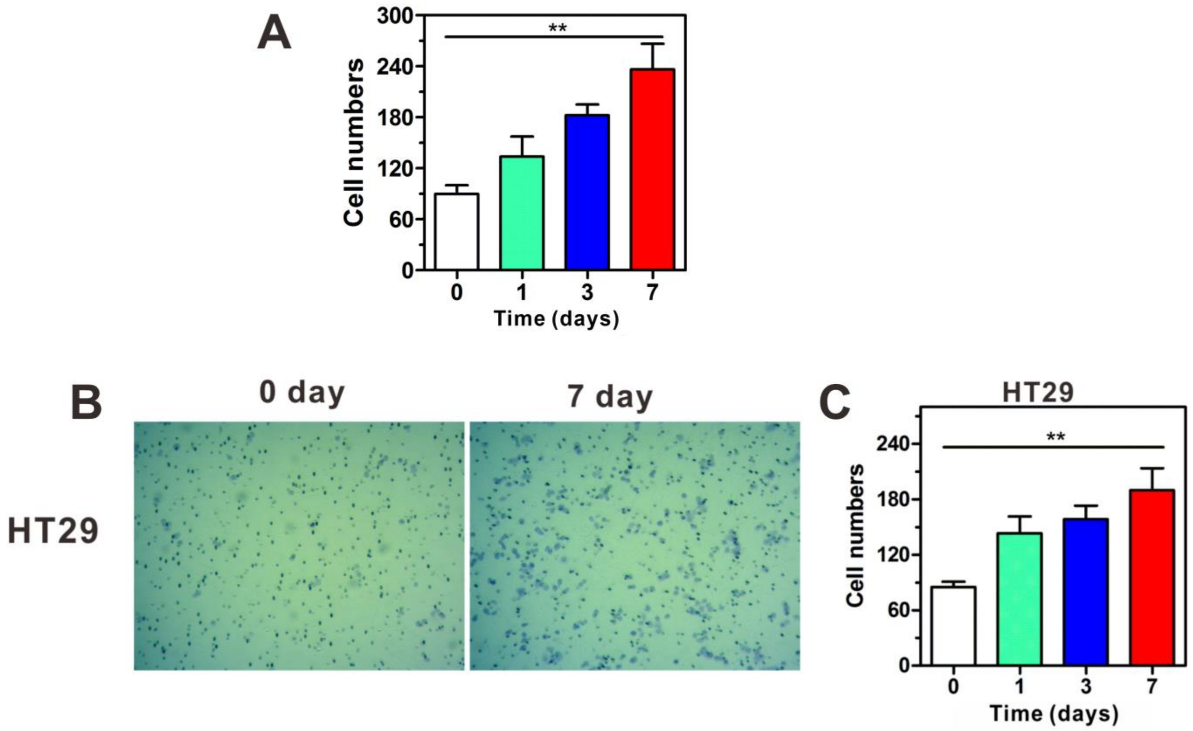

Supplementary Figure 3. The cell invasion ability was determined by transwell assay in HCT116 **(A)** and HT29 **(B and C)** cells treated with TGF-β for different durations (0, 1, 3, and 7 days). Data are the mean ± SD (n=3). $^{**}p < 0.01$ compared to the control group (0 days).

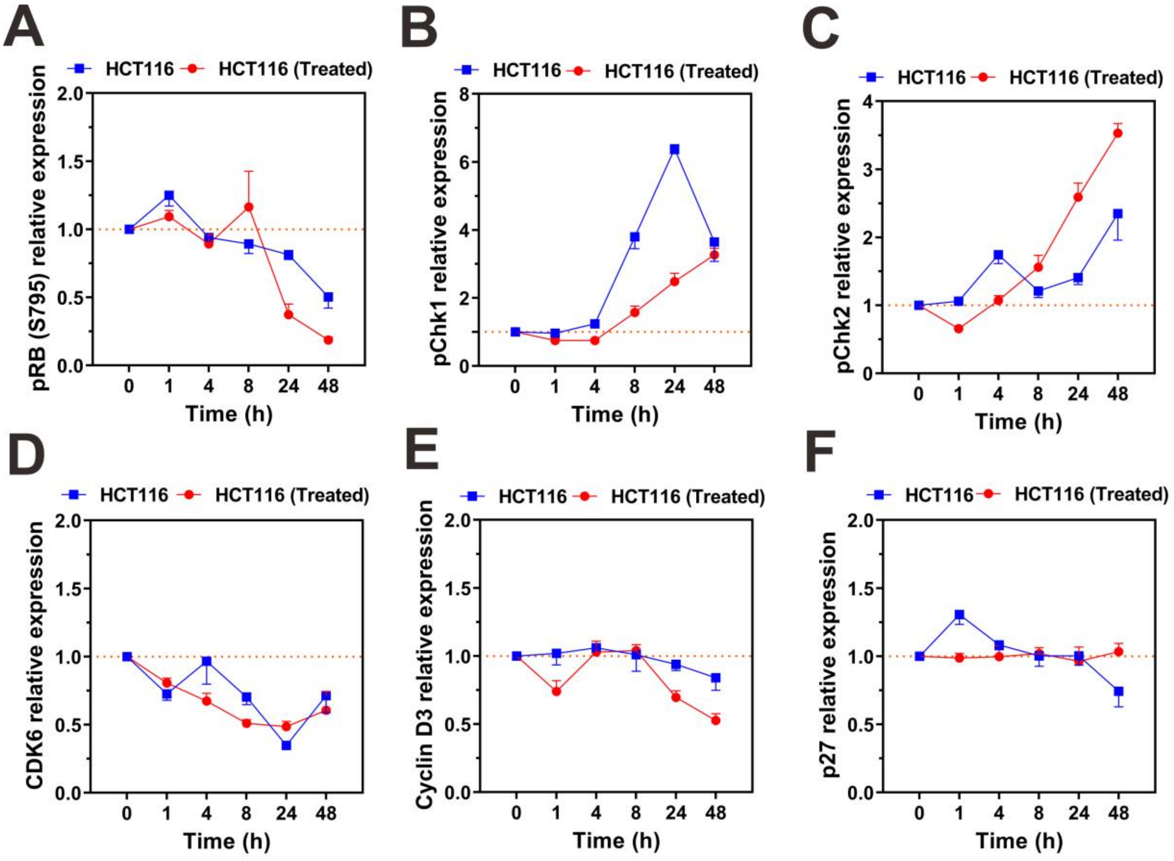

Supplementary Figure 4. The expression of cell cycle regulatory proteins in HCT116 and TGF-β-induced highly metastatic HCT116 (H-HCT116) cells with inhibition of oxidative phosphorylation (OXPHOS). Band intensity were quantified by using Image Lab analysis software and calculated as ratio of control group (0 h). HCT116 (Treated) indicates H-HCT116 cells. Data are the mean ± SD (n=3).
